# Anisotropic topographies restore endothelial monolayer integrity and promote the proliferation of senescent endothelial cells

**DOI:** 10.3389/fcvm.2022.953582

**Published:** 2022-10-05

**Authors:** Vasileios Exarchos, Sebastian Neuber, Heike Meyborg, Costanza Giampietro, Nafsika Chala, Silvia Moimas, Hristian Hinkov, Friedrich Kaufmann, Francesca M. Pramotton, Katrin Krüger, Hector Rodriguez Cetina Biefer, Nikola Cesarovic, Dimos Poulikakos, Volkmar Falk, Maximilian Y. Emmert, Aldo Ferrari, Timo Z. Nazari-Shafti

**Affiliations:** ^1^Cardiosurgical Research Group, Department of Cardiothoracic and Vascular Surgery, German Heart Center Berlin, Berlin, Germany; ^2^Translational Cardiovascular Regenerative Technologies Group, BIH Center for Regenerative Therapies, Berlin Institute of Health at Charité—Universitätsmedizin Berlin, Berlin, Germany; ^3^Experimental Continuum Mechanics, Empa Swiss Federal Laboratories for Materials Science and Technology, Dübendorf, Switzerland; ^4^Department of Mechanical and Process Engineering, Institute for Mechanical Systems, ETH Zürich, Zurich, Switzerland; ^5^Laboratory of Thermodynamics in Emerging Technologies, Department of Mechanical and Process Engineering, ETH Zürich, Zurich, Switzerland; ^6^Clinic for Cardiovascular Surgery, Charité—Universitätsmedizin Berlin, Berlin, Germany; ^7^Department of Cardiac Surgery, City Hospital of Zürich, Site Triemli, Zurich, Switzerland; ^8^Department of Health Sciences and Technology, ETH Zürich, Zurich, Switzerland; ^9^Department for Cardiovascular and Thoracic Surgery, German Heart Center Berlin, Berlin, Germany; ^10^Institute for Regenerative Medicine, University of Zurich, Zurich, Switzerland; ^11^BIH Biomedical Innovation Academy, BIH Charité (Junior) (Digital) Clinician Scientist Program, Berlin Institute of Health at Charité—Universitätsmedizin Berlin, Berlin, Germany

**Keywords:** endothelial cells, monolayer integrity, proliferation, topography, anisotropy, senescence, telomere, aging

## Abstract

Thrombogenicity remains a major issue in cardiovascular implants (CVIs). Complete surficial coverage of CVIs by a monolayer of endothelial cells (ECs) prior to implantation represents a promising strategy but is hampered by the overall logistical complexity and the high number of cells required. Consequently, extensive cell expansion is necessary, which may eventually lead to replicative senescence. Considering that micro-structured surfaces with anisotropic topography may promote endothelialization, we investigated the impact of gratings on the biomechanical properties and the replicative capacity of senescent ECs. After cultivation on gridded surfaces, the cells showed significant improvements in terms of adherens junction integrity, cell elongation, and orientation of the actin filaments, as well as enhanced yes-associated protein nuclear translocation and cell proliferation. Our data therefore suggest that micro-structured surfaces with anisotropic topographies may improve long-term endothelialization of CVIs.

## Introduction

**FIGURE 1:**
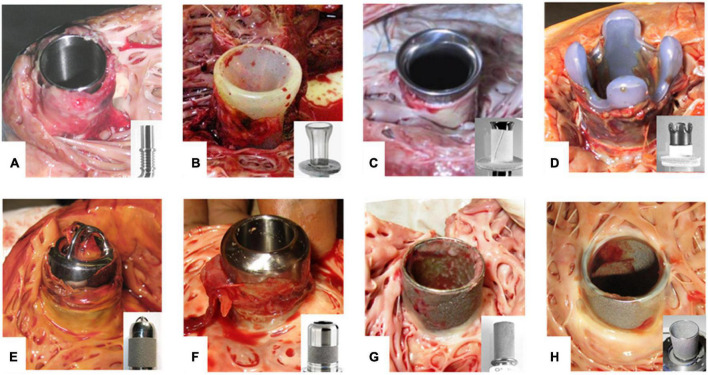
Thrombus formation at LVAD inflow cannulas. **(A–H)** Inflow cannulas of various LVADs after explanation. Cannulas without any textured surface are prone to thrombus development of unhindered growth **(A,B)**. Textured cannulas show scar tissue at the well healed myocardial wound of the entry site covering the lower section with a textured surface consisting of woven polyester cuff **(C,D)** or sintered titanium microspheres **(E–H)**. Intracaval sintered parts of the cannula frequently are covered by fibrin layers of various thickness **(G,H)**. Especially at intraluminal parts these layers may form patchy structures or increased thickness with the risk of constricting the flow path or peeling off. Thrombus formation often is originating from the transition to the polished tip with uncontrolled growth. EC lining should prevent thrombus formation and propagation. **(A)** DuraHeart (Terumo Heart, Inc., Ann Arbor, MI, USA), totally polished titanium inflow cannula. **(B)** VentrAssist (Ventracor, Chatswood, NSW, Australia), smooth silicone cannula without texturing. **(C)** DeBakey pump (MicroMed Cardiovascular, Inc., Houston, TX, USA), lower part textured with Dacron cuff, polished titanium cannula. **(D)** INCOR (BerlinHeart, Berlin, Germany) with Dacron cuff and smooth silicone tip. **(E)** Jarvik-2000 (Jarvik Heart, New York, NY, USA). **(F)** HVAD (Medtronic Inc., USA) inflow cannula featuring about 50% sintered surface length. **(G)** Heartmate-II (Abbott, Pleasanton, CA, USA). **(H)** HeartMate-3 with totally sintered cannula sparing about 1 mm at the cannula brim.

Despite technological advances, the use of cardiovascular implants (CVIs) such as left ventricular assist devices (LVADs) or heart valves is associated with adverse events, including thromboembolic complications that may lead to stroke or systemic embolism (1, 2). Thrombus formation occurs predominantly on foreign or xenograft surfaces exposed to low flow, e.g., the inflow cannula of LVADs (Figure 1) or the aorta facing surfaces of bioprosthetic or mechanical heart valves (3–5). Hence, the formation of a monolayer of endothelial cells (ECs) on these surfaces could reduce the contact area between blood and synthetic components, thereby improving the hemocompatibility of CVIs and thus reducing thromboembolic events (6, 7).

In general, there are two strategies for achieving endothelialized CVIs: (i) *in vitro* endothelialization and (ii) *in vivo* endothelialization. *In vitro* endothelialization requires the *ex vivo* seeding of ECs on CVI surfaces from either autologous vascular tissue (e.g., saphenous vein) or allogeneic sources (e.g., umbilical cord), whereas *in vivo* endothelialization involves EC migration from the intact endothelium or by adhesion, proliferation, and differentiation of circulating endothelial progenitors (8). Both approaches require chemical and/or biological functionalization of the luminal surfaces of CVIs to support cell recruitment and adhesion (7, 9). For example, surface textures produced by nanotechnology modulate the dynamics of cell protrusion (i.e., the establishment of initial adhesions and their interaction with the cytoskeleton), thus contributing to fundamental steps in the initial phase of the endothelialization process (10, 11).

Overall, patients who could benefit from CVI endothelialization usually have multiple co-morbidities and are of old age. However, these factors are directly linked to premature EC inflammatory and replicative senescence (RS), a major challenge in the formation of intact EC monolayers when using autologous cells (12). In general, senescence is a cellular state of hibernation characterized by a typical irreversible cell cycle arrest, coupled with changes at the transcriptional, metabolic, and secretory levels as well as a modified cell morphology. As the percentage of senescent ECs in a monolayer will greatly impact its functionality (13, 14), it is necessary to develop strategies that promote the maintenance of a functional EC monolayer not only for young and healthy ECs, but also for aged and senescent ECs. Biochemical changes during the transition from proliferating to senescent ECs are well characterized, but associated biomechanical changes are relatively unknown. As it has been observed that micro-structured surfaces with gratings can enhance EC alignment and improve the integrity of cell-to-cell junctions (15), we hypothesized that surfaces with gratings may not only improve the biomechanical properties of senescent ECs, but also increase their replication capacity.

## Materials and methods

### Cell isolation and cell culture

Human umbilical cord vein endothelial cells (HUVECs) and human umbilical cord mesenchymal stems cells (HUMSCs) were isolated from umbilical cords that were obtained from the Department of Obstetrics and Gynecology at the Charité-Universitätsmedizin Berlin. The collection and use of the samples was approved by the institutional ethics board under application number EA2/178/13. In total, three different healthy donors were included in this study. All cells were cultured in Medium 200 (Thermo Fisher Scientific, Waltham, MA, USA) supplemented with low-serum growth supplement (Thermo Fisher Scientific, Waltham, MA, USA) at 37°C with 5% CO_2_. The final concentrations of supplements were 2% fetal bovine serum, 1 μg/ml hydrocortisone, 10 ng/ml human epidermal growth factor, 3 ng/ml basic fibroblast growth factor, and 10 μg/ml heparin. During *in vitro* expansion, cells were either seeded on plates at a density of 20,000 cells/cm^2^, coated with 1.5% gelatine solution (Millipore Sigma, Burlington, MA, USA) or on a surface with gratings (described below) at a density of 5 × 10^3^ cells/cm^2^ coated with glutaraldehyde cross-linked gelatin to increase cell adhesion. When cells were grown on the gratings, the medium was supplemented with a higher serum concentration of 10% fetal bovine serum. For both conditions, the medium was changed every 2–3 days and the cells were passaged at 80–90% confluence.

### Flow cytometry analysis

Cells were harvested after reaching 70–80% confluence and approximately 1 × 10^6^ cells were suspended in 1 ml flow cytometry buffer containing phosphate-buffered saline (PBS) with 5 mM EDTA, 25 mM HEPES, 1% fetal bovine serum, and 1% penicillin/streptomycin at pH 7.6. After centrifugation at 300 × g for 5 min, the cell pellets were incubated with the following antibodies for 30 min at 4°C in the dark: anti-human CD31/PE-Cy7 (clone WM59, BioLegend, San Diego, CA, USA), anti-human CD105/PE (clone 43A3, BioLegend, San Diego, CA, USA), or anti-human CD90/FITC (clone 5E10, Invitrogen, Carlsbad, CA, USA), each at a concentration of 5 μg/ml in PBS containing 5% bovine serum albumin (BSA). The samples were washed twice with flow cytometry buffer, fixed with flow cytometry buffer supplemented with 0.5% paraformaldehyde (PFA) and stored at 4°C until measurement using a FACS Canto II Flow Cytometer (BD Biosciences, USA). Analysis was performed using FlowJo version 10.6.1 (Tree Star, Ashland, OR, USA).

### Cell proliferation and population doubling analysis

Cell proliferation rate was determined using a cell proliferation kit (Click-iT EdU Cell Proliferation Kit for Imaging, Alexa Fluor 555 dye) according to the manufacturer’s instructions. Images were taken with the Keyence BZ-X710 fluorescence microscope (Keyence, Itasca, IL, USA) and the number of 5-ethynyl-2’-deoxyuridine (EdU)-positive cells was analyzed using Imaris software (version 9.6.0, Oxford Instruments, Abingdon, UK).

The population doubling time (PDT) was calculated using the following equation: PDT = t⋅log2/[log(Nf)-log(N0)], where t is the culture duration, Nf is the number of harvested cells and N0 is the number of seeded cells.

### Senescence-associated β-galactosidase assay

Senescence-associated β-galactosidase (SA-β-gal) activity was measured using a histochemical senescence detection kit (Cell Signaling Technology, MA, USA, catalog no. 9860) according to the manufacturer’s instructions. Briefly, 2–3 × 10^5^ HUVECs per well were seeded in a 48-well plate and cultured for 24 h. Cells were washed twice with PBS at pH 7.4 and fixed with the fixative solution for 15 min at room temperature. After two washes with PBS, cells were incubated at 37°C without CO_2_ supply in freshly prepared SA-β-gal staining solution. After 12 h, the reaction was stopped by removing the SA-β-gal staining solution and cells were washed twice with PBS. To ensure a representative count, each tissue culture dish was divided into four quarters and at least one image was obtained from each quarter. The percentage of SA-β-gal-positive cells was determined by counting all SA-β-gal-positive cells under bright field illumination. The total number of cells was determined by 4′, 6-diamidino-2-phenylindole (DAPI) counterstaining (0.1 μg/ml in PBS, 15 min) and subsequent fluorescence microscopy (BZ-X710, Keyence, USA). Data analysis was performed using ImageJ software (version 1.52p, National Institutes of Health, USA) (16).

### Telomere length measurement

Relative telomere length was measured using a modified version of the monochrome multiplex quantitative polymerase chain reaction (PCR) protocol as previously described (17, 18). In brief, total DNA was isolated from HUVECs using Direct PCR Lysis Buffer (Viagen Biotech, Inc., CA, USA) and the DNA equivalent of 100,000 cells was added to each PCR reaction. The PCR reaction was performed in technical triplicates using a LightCycler 480 PCR system (Roche, Basel, Switzerland). A serial dilution of DNA from low passage HUVECs (passage 2) ranging from 10 to 1,200 pg was included in each assay to create a standard curve for sample DNA quantification. Reaction mixtures (10 μl) contained 20 mM Tris-HCl (pH 8.4), 50 mM KCl, 3 mM MgCl_2_, 0.2 mM of each deoxyribonucleotide triphosphate, 1 mM dithiothreitol, 1 M betaine (Affymetrix, Santa Clara, CA, USA), 0.5 × SYBR Green I (Thermo Fisher Scientific, Waltham, MA, USA), 0.1875 U Platinum Taq DNA polymerase (Thermo Fisher Scientific, Waltham, MA, USA), 0.0625 × Titanium Taq DNA polymerase (Clontech Laboratories, Mountain View, CA, USA), and 900 nM of each primer (telg, telc, hbgu, and hbgd). Primer sequences are given in Cawthon (19). Amplification conditions were as follows: 2 min at 95°C, followed by 6 cycles for 15 s at 95°C, and 15 s at 49°C, followed by 40 cycles for 15 s at 95°C, 10 s at 62°C, and 15 s at 72°C with signal acquisition, followed by 15 s at 84°C and 10 s at 88°C with signal acquisition. Roche LightCycler 480 software (version 1.5, Roche, Basel, Switzerland) was used to generate standard curves and to calculate DNA concentrations of telomeres and single-copy genes for each sample. The relative telomere lengths (T/S ratios) were calculated for each sample replicate.

### Cyclic olefin copolymer-slide preparation and flow experiments

Cyclic olefin copolymer (COC)-coated surfaces were obtained from Ibidi GmbH (Gräfelfing, Germany) and cut into rectangular slides of 3 cm × 1.5 cm. The slides were cleaned with 70% ethanol, washed three times with PBS, and coated with glutaraldehyde cross-linked gelatin to increase cell adhesion (20). In detail, COC slides were incubated with 1.5% gelatin solution for 1 h at 37°C, followed by cross-linking with 2% glutaraldehyde solution (Sigma Aldrich, St. Louis, MO, USA) for 15 min at room temperature. Glutaraldehyde was removed and the COC slides were incubated with 70% ethanol for 1 h at room temperature. After five washes with PBS followed by overnight incubation with PBS containing 2 mM glycine, the COC slides were washed five times with PBS and stored at 4°C prior to cell seeding.

All flow experiments were performed using a specifically designed parallel plate flow chamber to apply a constant shear stress to the monolayer of HUVECs (10). The shear stress (τ) applied to the monolayers can be expressed as a function of the channel dimension (width, w and height, h), medium property (viscosity, μ) and volumetric flow rate (Q) using the following formula for wall shear stress (WSS) in a rectangular channel: τ = (6⋅Q⋅μ)/(w⋅h^2^). While the channel dimension and medium property were fixed in our experimental setup (*w* = 20 mm, *h* = 0.3 mm, μ = 8.4 × 10^–4^ Pa⋅s), the flow rate was controlled using a peristaltic roller pump (Model 66, Harvard Apparatus, Holliston, MA, USA) to apply a WSS of 1.4 Pa to the endothelial monolayer. A compliance element was inserted between the roller pump and the flow chamber to dampen flow pulsation. The flow chamber was connected to the pump with Tygon tubing (Radnor, PA, USA) certified for low cytotoxicity. The COC slides containing a cell monolayer with a density of 4–5 × 10^4^ cells/cm^2^ was placed in the flow chamber, which was then filled with medium and connected to the hydraulic circuit. The flow bioreactor and the medium reservoir were allocated in a chamber in which the environment was maintained at 37°C and 5% CO_2_. The cell monolayer was then exposed to the flow generating the desired shear stress conditions for 16 h.

### Preparation of surfaces with anisotropic topography

To produce surfaces with gratings, template wafers with gratings of 1 μm depth and height were first fabricated at the cleanroom facilities of the Binnig and Rohrer Nanotechnology Center by a standard nanoimprint lithography process as previously reported (10). At the end of the fabrication process, the wafers were rinsed with acetone and 2-propanol, followed by immersion in dimethyldichlorosilane for 15 min to facilitate silanization. To transfer the same geometries to silicone elastomers, the template wafer was used as a direct form. Polydimethylsiloxane (PDMS) components A and B (Sylgard 184 Silicone Elastomer, Dow Chemical Company, MI, USA) were mixed in a ratio of 1:10 and poured onto the template, while elastomeric substrates were mixed in a ratio of 1:10. Silicone membranes with the desired topography were then cured for 1 h at 80°C and carefully removed from the wafer. For a direct comparison within each experiment, silicones with unstructured (flat) and topographically modified (grating) surfaces were produced. Up to six imprinted PDMS substrates were sealed in a well of a 6-well plate and cleaned by treatment with ethanol overnight. After washing three times with PBS, the surfaces were coated with glutaraldehyde cross-linked gelatin as described above and stored at 4°C prior to cell seeding.

### Immunofluorescence microscopy

At the end of each experiment, cells were fixed with 4% paraformaldehyde (PFA) for 20 min, washed with PBS, permeabilized with 0.5% Triton-X100 for 10 min and blocked with 5% BSA in PBS for 1 h. For the biomechanical characterization of senescent HUVECs under flow conditions, the samples were incubated with the following primary antibodies overnight at 4°C: rabbit anti-vascular endothelial cadherin (VEC) antibody (catalog no PA5-19612, Abcam, Cambridge, UK, 1:100) and rabbit anti-GOLM1 antibody (catalog no HPA010638, Sigma Aldrich, St. Louis, MO, USA, 1:100). After washing three times with PBS, cells were incubated with Alexa Fluor 568-labeled donkey anti-rabbit antibody (catalog no A-21206, Thermo Fisher, Waltham, MA, USA, 1:200) for 1 h at room temperature protected from light. To visualize cytoskeleton, F-actin fibers were directly stained with Alexa Fluor 555-labeled phalloidin (catalog no A34055, Thermo Fisher, Waltham, MA, USA, 1:200). For the biomechanical characterization of senescent HUVECs after cultivation on anisotropic topography with gratings, in addition to the staining for VEC protein and F-actin fibers, the samples were incubated overnight at 4°C with mouse anti-yes-associated protein (YAP) primary antibody (catalog no sc-101199, Santa Cruz, Dallas, TX, USA, 1:100), and after washing three times with PBS, the cells were incubated with Alexa Fluor 555-labeled donkey anti-mouse antibody (catalog no A31570, Thermo Fisher, Waltham, MA, USA, 1:100). To visualize focal adhesions (FAs), the samples were stained with the following primary antibodies overnight at 4°C: mouse anti-paxillin antibody (catalog no 610051, BD Bioscience, USA, 1:100) and rabbit anti-p-paxillin Tyr118 antibody (catalog no 2541S, Cell Signaling Technology, USA, 1:100). After washing three times with PBS, the cells were incubated with the following secondary antibodies: Alexa Fluor 488-labeled donkey anti-mouse antibody (catalog no A-21202, Thermo Fisher, Waltham, MA, USA, 1:100) and Alexa Fluor 555-labeled donkey anti-rabbit antibody (catalog no A-32794, Thermo Fisher, Waltham, MA, USA, 1:100), respectively. After washing three times in PBS, samples were mounted with medium containing DAPI (0.1 μg/ml). Images were taken with the Keyence BZ-X710 fluorescence microscope (Keyence, Itasca, IL, USA) and analyzed using ImageJ (version 1.52p) and MATLAB (version 7.0, MathWorks, USA) (16).

### Image analysis

To quantify the number of phosphorylated histone H2AX (γ-H2AX) foci per nucleus, images were analyzed and processed using the FoCo algorithm (21). A nuclear mask was created based on the DAPI fluorescent signal using MATLAB (MathWorks, USA) and ImageJ (version 1.52p, National Institute of Health, USA). The distribution of the γ-H2AX signal in nucleus was then analyzed to count the number of foci. Cells were categorized in three groups based on the number of detected foci (0, 1–3, and > 4). Quantitative analysis of FAs in the peripheral region of the cell was based on a previously reported protocol (22). Both the number of FAs divided to the cell perimeter, known as linear FA density, as well as the FA size were obtained by analyzing the number of FAs after excluding the nuclear and perinuclear fields in the cell center. To assess the polarization of HUVECs in relation to the flow, the nucleus and the Golgi apparatus of cells were marked and saved as separate files using the Imaris software. The position of the nucleus was calculated in relation to the Golgi apparatus using MATLAB software. The connectivity index (CI) was calculated from the average skewness (Rsk) of the VEC signal profile along four directions according to the following formula: CI = average [Rsk(north), Rsk(west), Rsk(south), Rsk(east)]. Each CI value was normalized according to the maximum value. CI values close to 1 indicate a monolayer with a fully preserved network of cell-to-cell junctions typical for long confluent EC monolayers, while values close to 0 indicate a diffuse VEC signal in the cytoplasm, which is typical for EC monolayers with disassembled or immature junctions. A connectivity threshold was established by measuring the VEC-CI in recently confluent monolayers, i.e., after 12–16 h of cultivation (23, 24).

The tortuosity of endothelial adherent junctions was obtained from the VEC signal. Briefly, the VEC adherent junctions were traced in ImageJ (version 1.52p), and for each junction, the contour length of the junction, *L*, and the end-to-end distance, *D*, were measured. The tortuosity was then computed as the ratio of *L*/*D*. Tortuosity index values close to 1 indicate a straight junction, while higher values indicate highly convoluted junction topology (15). A measure of average cell orientation was obtained from the VEC trace or the F-actin signal using the freehand selection tool of ImageJ. In detail, the cell profile was manually drawn and the cell orientation angle along with additional shape features of the cell were obtained using the “Fit ellipse” option in the “Measurements” tool. The range of possible alignment angles between cells and the direction of flow was 0–90 degrees; a value close to 0° indicates parallel alignment, a value close to 45° indicates no alignment and a value close to 90° indicates perpendicular alignment. The aspect ratio of cells was calculated as the ratio of the length of the major axis to the length of the minor axis.

To quantify the level of cytoplasmic-to-nuclear YAP translocation, the ratio between the cytoplasmic and nuclear YAP mean signal intensity was calculated using MATLAB (MathWorks, USA) and ImageJ (version 1.52p, National Institute of Health, USA) (25).

### Statistical analysis

GraphPad Prism 9.0 (San Diego, CA, USA) was used for performing data analysis and generating graphs. The comparison of population means was done using a two-way analysis of variance and a *p*-value of less than 0.05 was considered significant. All quantitative measurements reported are expressed as average values plus standard errors of the mean. Analysis was always based on three or more independent experiments, as specified in each figure legend. For box plots, the size depicts the measured standard error of the mean and the whiskers indicate 5% and 95% confidence intervals.

## Results

### Replicative senescence in human umbilical cord vein endothelial cells compromises monolayer integrity

**FIGURE 2:**
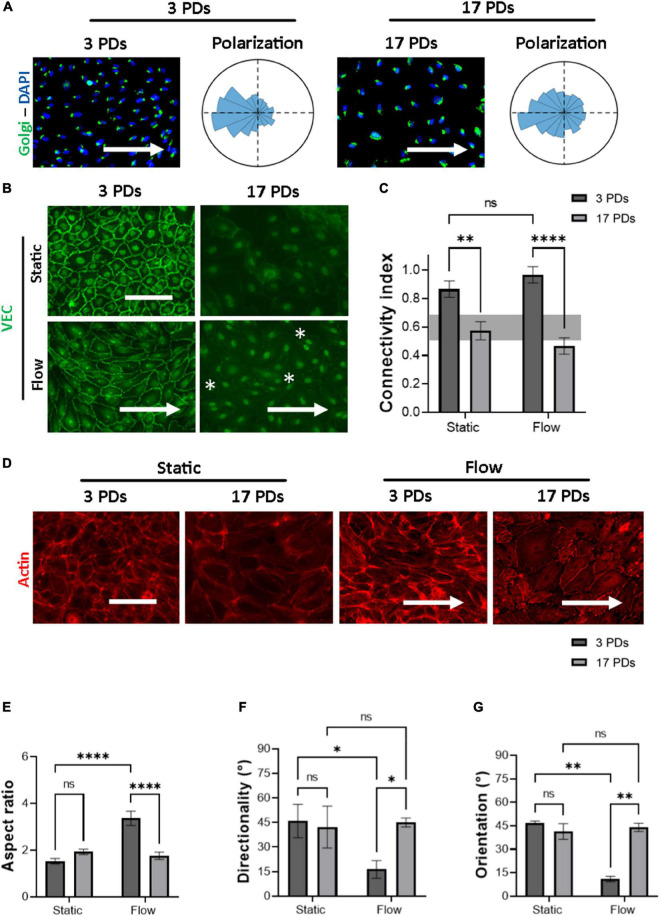
Highly passaged HUVECs lose their monolayer integrity under physiological flow of 1.4 Pa. Representative immunofluorescence microscopy images for analysis of HUVECs cultured under flow conditions generating a WSS value of 1.4 Pa for 16 h; **(A)** cells stained with an antibody against the Golgi apparatus (green), the nuclei were stained with DAPI (blue). Histograms indicate the assessment of cell polarity. **(B)** Cells stained with an antibody against VEC (green), white asterisks indicate signs of monolayer disruption. **(C)** Measure of normalized VEC-CI values in non-senescent (3 PDs) and senescent (17 PDs) HUVEC monolayers grown under static and flow conditions with a WSS value of 1.4 Pa. **(D)** Cells stained with an antibody against Phalloidin (red). In **(A,B,D)** arrows indicate the direction of flow. Scale bars, 100 μm. **(E)** Aspect ratio of cells defined as the ratio of major- to minor-axis length. **(F)** Average directionality of actin filaments in degrees according to the direction of the flow. **(G)** Average cell orientation in degrees according to the direction of the flow. In **(C,E–G)**, data represent the mean and standard error of the mean of 3 donors with more than 100 cells per condition. *p < 0.05; **p ≤ 0.01; ****p ≤ 0.0001; ns, not significant.

In our study, we used HUVECs that were freshly isolated form umbilical cords and showed a typical cobblestone morphology and classical endothelial surface markers such as CD31 and CD105 under *in vitro* conditions on population doubling level (PDL) 3 (Supplementary Figure 1). RS was induced by extended *in vitro* culture, resulting in cells with significant formation of DNA double-strand breaks, increased senescence-associated β-galactosidase (SA-β-gal) activity, and significantly shortened relative telomere lengths (Supplementary Figure 2), three typical markers of RS (26, 27). Furthermore, *in vitro* cell expansion affected not only cell growth characteristics such as PDT and replication capacity, but also the cell density of the EC monolayer and cell morphology, as also described by others (Supplementary Figures 2, 3) (28, 29). Next, we characterized the following biomechanical properties of young [3 population doublings (PDs)] and senescent (17 PDs) HUVECs that are considered to play an important role in the formation of a stable and functional monolayer on foreign surfaces under physiological flow:

a. the polarization of cells through the interplay between the Golgi apparatus and the nucleus (Figure 2A).

b. the formation of mature cell-to-cell adherens junctions (AJs) according to the distribution of VEC signal across the cell outer membrane, measured through the CI (Figures 2B,C), and
c. the orientation and elongation of ECs according to the actin filament reorganization (Figures 2D–G),

Under flow conditions, both low-passage HUVECs (3 PDs) and high-passage HUVECs (17 PDs) achieved counterflow polarization (Figure 2A). Under both static and flow conditions, immunostaining of VEC protein revealed a significant loss in connectivity, ranging from 0.9 ± 0.05 (3 PDs) to 0.6 ± 0.05 (17 PDs) under static conditions (*p* = 0.001) and from 1.0 ± 0.1 (3 PDs) to 0.5 ± 0.03 (17 PDs) under flow conditions (*p* = 0.0001), respectively (Figures 2B,C). At 3 PDs, HUVECs aligned their shape along the direction of flow, whereas high-passage HUVECs lost the ability to form a monolayer with well-organized elongated and aligned actin stress fibers (Figure 2D). The aspect ratio changed significantly for HUVECs at low passage from 1.5 ± 0.1 under static conditions to 3.3 ± 0.3 under flow conditions (*p* = 0.0001), but was similar to HUVECs at high passage with values of 1.96 ± 0.1 under static conditions and 1.76 ± 0.1 under flow conditions (Figure 2E). Similarly, the average directionality of actin filaments for low-passage HUVECs was 45 ± 8° under static conditions and 16 ± 4° under flow conditions (*p* = 0.01). At 17 PDs, there was no significant difference in directionality under both static and flow conditions (Figures 2D,F). Our data also showed that low-passage HUVECs aligned with the direction of flow, a property that was lost at high passage (11 ± 1.7° vs. 44 ± 2°, respectively; *p* = 0.004) (Figure 2G). In conclusion, highly passaged HUVECs maintained their ability to achieve counterflow polarization. However, they lost their connectivity as well as their ability to elongate and orient in response to actin filament reorganization under parallel flow with physiological WSS.

### Cultivation on a micro-structured surface with gratings restores the monolayer integrity of senescent human umbilical cord vein endothelial cells

**FIGURE 3:**
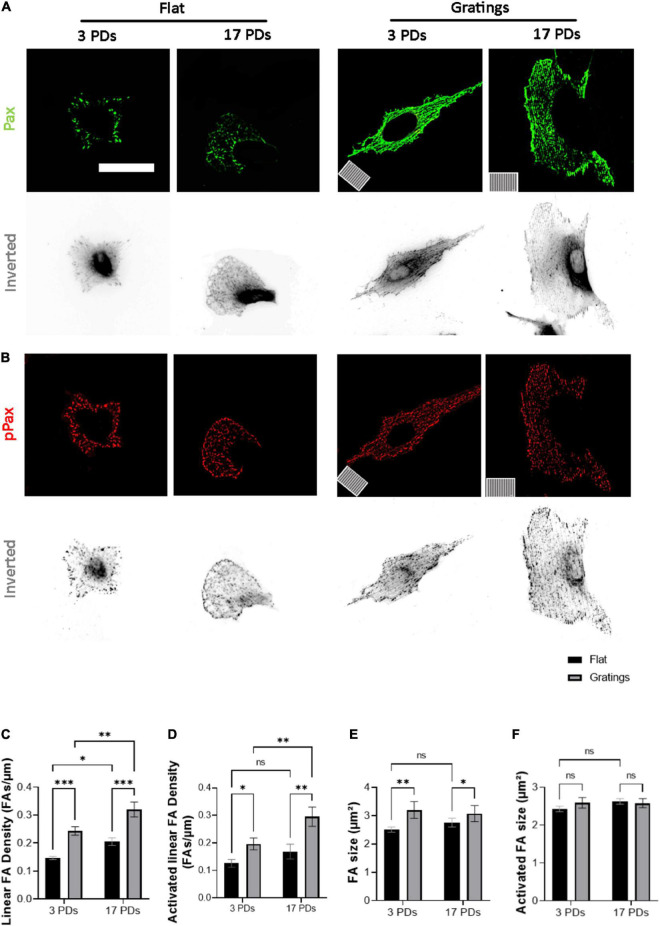
Gratings enhance the establishment of FA and promote FA activation. **(A,B)** Representative immunofluorescence analysis of HUVECs cultured under static conditions on a flat surface and a micro-structured surface with gratings; cells were stained with **(A)** an antibody against paxillin (Pax, green) and **(B)** an antibody against phosphorylated paxillin (pPax, red). The orientation of the gratings is indicated on the panels in the lower left corner. Scale bars are 100 μm. **(C)** Linear density of FA analyzed in HUVECs after 3 and 17 PDs on a flat surface and a surface with gratings. The number of FAs is normalized to the cell perimeter. **(D)** Linear density of activated FAs. **(E)** FA size after 3 and 17 PDs. **(F)** Activated FA size after 3 and 17 PDs. Data represent the mean and standard error of the mean of 3 donors with more than 360 FAs per condition. *p < 0.05; **p ≤ 0.01; ***p ≤ 0.001; ns, not significant.

**FIGURE 4:**
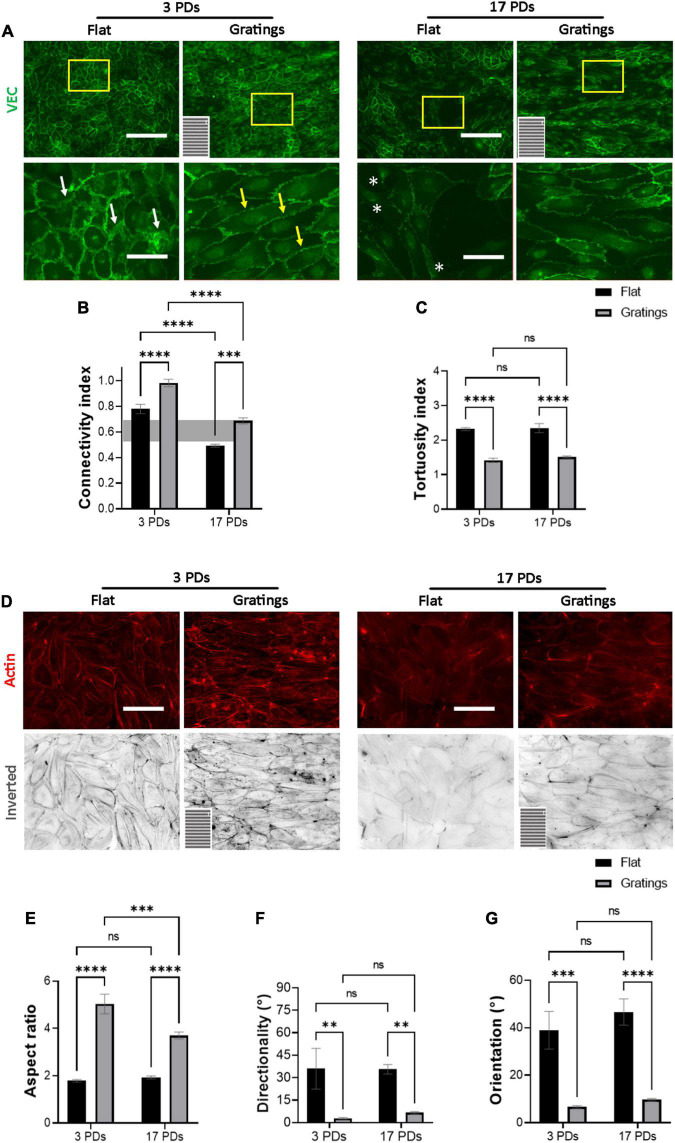
Gratings restore the monolayer integrity of HUVECs. Representative immunofluorescence microscopy images for the analysis of monolayers of HUVECs after 3 and 17 PDs cultured either on a flat surface or on a micro-structured surface with gratings. **(A)** Cells stained with an antibody against VEC (green), white arrows indicate zipper-like junctions on a flat surface, while yellow arrows indicate linear junctions on a surface with gratings. White asterisks indicate signs of monolayer disruption on the flat surface. **(B)** Measure of normalized VEC-CI values in non-senescent (3 PDs) and senescent confluent monolayers (17 PDs) grown on a flat surface or on a surface with gratings. A horizontal gray bar indicates the threshold value that defines differentiated ECs and was measured in recently confluent endothelial monolayers after 3 PDs. **(C)** Tortuosity index in non-senescent (3 PDs) and senescent confluent monolayers (17 PDs) of ECs grown on a flat surface or on a surface with gratings. **(D)** Cells stained with an antibody against phalloidin (red). The orientation of the gratings is indicated on the panels in the lower left corner. Scale bars are 200 μm **(A)** and 100 μm **(B)**. **(E)** Aspect ratio of HUVECs defined as the ratio of major- to minor-axis length. **(F)** Average directionality of actin filaments in degrees relative to the orientation of the gratings. **(G)** Average cell orientation relative to the orientation of the gratings. In **(B,C,E–G)**, data represent the mean and standard error of the mean of 3 donors with more than 100 cells per condition. *p < 0.05; **p ≤ 0.01; ***p ≤ 0.001; ****p ≤ 0.0001; ns, not significant.

As it has been observed that micro-structured surfaces with gratings can enhance EC alignment and improve the integrity of cell-to-cell junctions (15), we produced surfaces with an anisotropic topography with gratings of 1 μm width and height (Supplementary Figure 4) and tested under static conditions whether these topographies have a positive impact on the biomechanical properties of both non-senescent and senescent HUVECs. At first, we investigated the cell anchoring to the surface (flat vs. grating) by imaging paxillin and its phosphorylated form as important regulators of FA assembly and disassembly (Figures 3A,B) (30). On the flat surface, senescent HUVECs (17 PDs) exhibited a significant increase in the linear density of inactivated FAs in relation to non-senescent HUVECs (3 PDs) from 0.15 ± 0.01 to 0.21 ± 0.01 FA/μm (Figure 3A left panel and Figure 3C, *p* = 0.04). The linear density of activated FAs was also increased significantly after 17 PDs, from 0.12 ± 0.01 to 0.17 ± 0.02 FA/μm (Figure 3B left panel and Figure 3D, *p* = 0.006). A concomitant increase in the size of inactivated and activated FAs was not observed (Figures 3E,F). Compared to cultivation on a flat surface, HUVECs cultured on gratings also displayed a significant increase in linear FA density from 0.15 ± 0.01 to 0.24 ± 0.01 FA/μm after 3 PDs and from 0.21 ± 0.01 to 0.32 ± 0.02 FA/μm after 17 PDs (Figure 3A right panel and Figure 3C, *p* = 0.01 for both conditions). Similarly, the activated linear FA density increased from 0.12 ± 0.1 to 0.19 ± 0.02 FA/μm after 3 PDs and from 0.17 ± 0.02 to 0.3 ± 0.03 FA/μm after 17 PDs (Figure 3D, *p* = 0.001 and *p* < 0.001 respectively) when cultured on a surface with gratings. Accordingly, the inactivated FA size increased from 2.51 ± 0.1 to 3.2 ± 0.2 μm^2^ after 3 PDs and from 2.6 ± 0.1 to 3.1 ± 0.2 μm^2^ after 17 PDs (Figures 3E,F, *p* = 0.008 and *p* = 0.03 respectively). In summary, our data suggests that both senescence and cultivation on topographies lead to formation and maturation of FAs in HUVECs. In addition, HUVEC monolayers cultured on the micro-structured surface with gratings adopted elongated shape with no evidence of monolayer disruption and formed mainly linear AJs parallel to the grating axis with tortuosity index values close to 1. In contrast, HUVECs cultured on the flat surface mainly formed zipper-like AJs with no preferred orientation and resulted in an increase in tortuosity index values after 3 PDs (1.41 ± 0.05 for a surface with gratings vs. 2.31 ± 0.03 on a flat surface, *p* < 0.0001) and after 17 PDs (1.51 ± 0.02 for a surface with gratings vs. 2.34 ± 0.1 on a flat surface, < 0.0001) (Figures 4A,C). To quantify further the improved cell elongation on gratings in relation to the flat surface (Figures 4A,D), analysis of aspect ratio, actin filament directionality and cell orientation were performed. HUVECs showed an increase in the aspect ratio from 1.8 ± 0.05 to 5 ± 0.4 (*p* < 0.001) at 3 PDs, as well as after 17 PDs from 1.9 ± 0.06 to 3.7 ± 0.14 (*p* < 0.001) (Figure 4E). Intriguingly, cultivation on the gratings improved elongation of both cell groups, but not in the same extent as non-senescent HUVECs, which displayed a higher extent of elongation (Figure 4E, aspect ratio on gratings 5 ± 0.4 after 3 PDs and 3.7 ± 0.14 after 17 PDs, *p* ≤ 0.001). The average actin filament directionality of HUVECs after 3 PDs was 36 ± 11° on the flat surface and 3 ± 0.04° on the gratings (*p* = 0.002). A similar change from baseline was observed for HUVECs after 17 PDs; the average directionality of actin filaments was changed from 36 ± 3° on a flat surface to 7 ± 1° on a surface with gratings (*p* = 0.005) (Figure 4F). This data is in line with measurements on the average cell orientation, namely 39 ± 8° on the flat surface and 6 ± 0.6° on the gratings (*p* < 0.001). A similar change was observed for HUVECs after 17 PDs: from 47 ± 6° on a flat surface to 10 ± 0.6° on a surface with gratings (*p* < 0.001) (Figure 4G). There was no significant difference of average cell orientation on either surface (Figure 4G). Overall, Figure 4 demonstrates that anisotropic topographies increase VEC-CI, promote formation of linear VEC AJs as well as alignment and orientation of HUVECs.

### Cultivation of human umbilical cord vein endothelial cells on gratings enhanced the cytoplasmic-to-nuclear translocation of yes-associated protein

**FIGURE 5:**
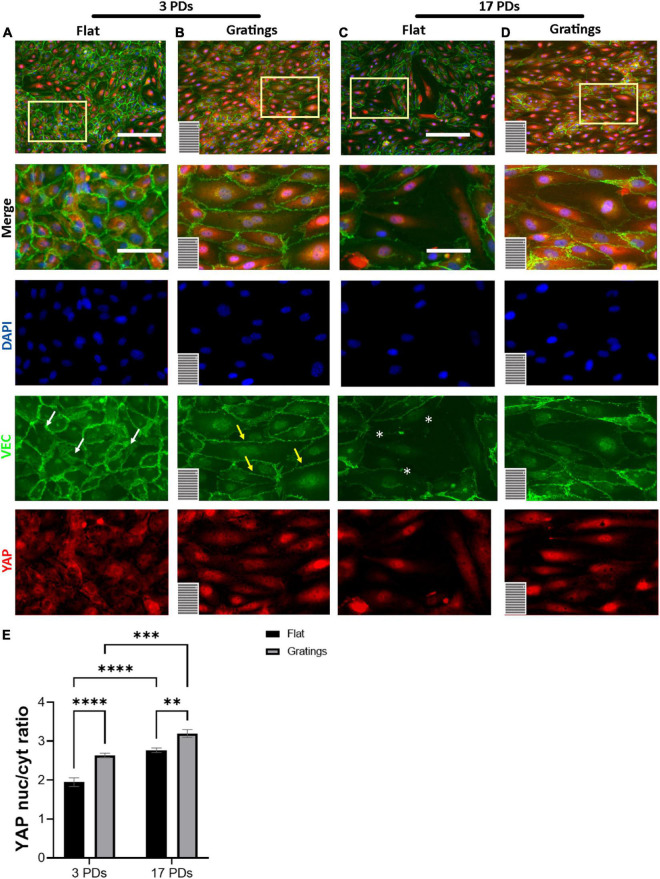
YAP nuclear translocation after HUVECs culture on gratings. **(A–D)** Immunofluorescence analysis of monolayers of HUVECs after 3 or 17 PDs cultured either on a flat surface or on a micro-structured surface with gratings. Cells were stained with an antibody against YAP (red), DAPI (blue), and VEC (green). The orientation of the gratings is indicated in the lower left corner. Yellow rectangles indicate the zoom-in areas. White arrows indicate zipper-like junctions on flat surface, while yellow arrows linear junctions on a surface with gratings. White asterisks indicate monolayer disruption on the flat surface. Scale bars, 200 μm; scale bars of zoomed images, 100 μm. **(E)** Quantification of YAP nuclear translocation as a ratio of YAP nuclear signal to YAP cytoplasmic signal. Data represent the mean and standard error of the mean 3 donors with more than 1,500 cells per condition. **p ≤ 0.01; ***p ≤ 0.001; ****p ≤ 0.0001.

It is known that surface-mediated cues may not only dictate FA formation and activation, cell-to-cell adhesion, and actin filament reorganization, but also cell proliferation (31). The latter is regulated through several mechanosensitive transcription factors such as the YAP (32). For this reason, we investigated under static conditions the impact of gratings on the activation of YAP. When cultured on flat surfaces, we observed that the majority of non-senescent HUVECs revealed a homogenous YAP signal equally distributed between the nucleus and the cytoplasm with a nucleus-to-cytoplasm ratio (NtCR) of 1.9 ± 0.1 (Figures 5A,E). In contrast, cultivation on the gratings resulted in a significant YAP translocation from the cytoplasm to the nucleus (NtCR of 2.6 ± 0.1, *p* < 0.001) (Figures 5B,E). Senescent HUVECs formed a heterogeneous monolayer on flat surfaces with an average NtCR of 2.8 ± 0.1 (Figure 5E). Cells with weak cell-to-cell contacts (Figure 5C) revealed an enhanced YAP signal in the nucleus compared to the cytoplasm. However, cells with high VEC signal at the cell junctions showed homogeneously distributed YAP signal in the nucleus and cytoplasm (Figures 5C,D). Interestingly, senescent HUVECs that were cultured on gratings formed a more homogenous monolayer with a high signal of YAP in the nucleus (NtCR of 2.8 ± 0.1 to 3.2 ± 0.1, respectively; *p* < 0.001) (Figures 5D,E).

### Cultivation of human umbilical cord vein endothelial cells on gratings enhances cell proliferation without increasing DNA damage or inducing an early onset of senescence

**FIGURE 6:**
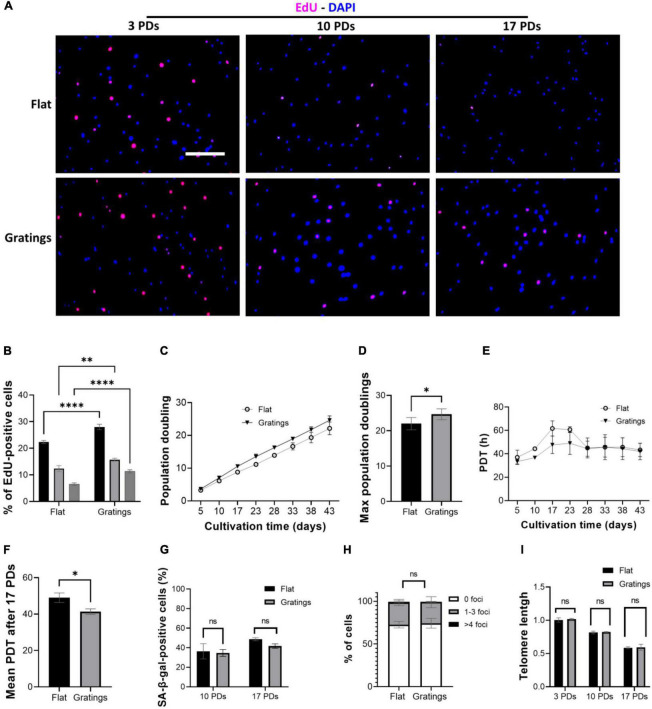
Gratings increase HUVEC proliferation without inducing an early onset of senescence or DNA damage. **(A)** EdU proliferation assay with cells after 3, 10, and 17 PDs, which were cultured on a flat surface (upper panel) or on a surface with gratings (lower panel). Blue, DAPI; Red, EdU. Scale bar, 400 μm. **(B)** Percentage of EdU-positive cells after 3, 10, and 17 PDs. **(C)** Cumulative growth curve during expansion of HUVECs isolated from three different umbilical cords on a flat surface and a micro-structured surface with gratings. **(D)** PDT of HUVECs during *in vitro* expansion on a flat surface and a micro-structured surface with gratings. **(E)** Maximum PDs achieved after cultivation on a flat surface or on a surface with gratings for a total period of 43 days. **(F)** Calculation of mean PDT after 17 PDs on a flat surface or on a surface with gratings. **(G)** Percentage of SA-β-gal-positive cells after 10 and 17 PDs on a flat surface or on a surface with gratings. **(H)** Percentage of cells presenting multiple γ-H2AX foci after 17 PDs on a flat surface or a surface with gratings. **(I)** Relative telomere length in relation to the number of PDs of HUVECs during *in vitro* expansion on a flat surface and a surface with gratings. Data represent the mean and standard error of the mean of 3 donors, for **(A,B)** more than 600 cells per condition, for **(E)** more than 2,500 cells per condition, for **(F)** more than 276 cells and for **(G)** more than 100,000 cells per condition were analyzed, *p < 0.05; **p ≤ 0.01; ****p ≤ 0.0001; ns, not significant.

These observations led us to hypothesize that modulating the surface topography could aid senescent cells not only improve their cytoskeletal organization, but also increase their proliferation rate and even enhance their replication capacity. Culture of HUVECs on a surface with gratings under static conditions not only resulted in a significantly increased YAP nuclear translocation compared to cultivation on a flat surface, but also significantly improved the proliferative capacity of HUVECs. In fact, Figures 6A,B show a higher number of EdU-positive HUVECs after short-term cultivation on gratings for 16 h. At 3 PDs, 22 ± 0.6% EdU-positive cells were detected on flat surfaces vs. 28 ± 1% EdU-positive cells on gratings (*p* = 0.003). At 10 PDs, the number of EdU-positive cells decreased to 12 ± 1.2% on flat surfaces vs. 16 ± 0.6% EdU-positive cells on gratings (*p* = 0.02). Finally at 17 PDs, 6 ± 0.5% of cells were EdU-positive on flat surfaces vs. 11 ± 0.6% on gratings (*p* = 0.006, Figures 6A,B). To investigate the positive impact of gratings on cell proliferation, we expanded HUVECs *in vitro* on a polydimethylsiloxane surface with gratings. After a total period of 43 days, HUVECs underwent cumulatively 22 ± 1 PDs on flat surface and 25 ± 1 PDs on the gratings (Figures 6C,D, *p* < 0.05). The mean PDT was 48 ± 3 h on the flat surface and 43 ± 2 h on the surface with the gratings (Figures 6E,F, *p* < 0.05). More interestingly, after 10 and 17 PDs, cells cultured on the flat surface as well as on the surface with gratings displayed the same level of SA-β-gal activity (Figure 6G). In addition, the enhanced proliferation on the gratings did not lead to increased accumulation of γ-H2AX and significant telomere attrition (Figures 6H,I). The percentage of cells with 1–3 γ-H2AX foci was 27 ± 3% on the flat surface and 26 ± 4% on the gratings (Figure 6H, *p* > 0.05). On both surfaces after 17 PDs, an up to 40% decrease of telomeres initial length was observed (Figure 6I). The relative telomere length decreased from 1.0 ± 0.03 to 0.58 ± 0.01 on the flat surface (*p* < 0.001) and from 1.1 ± 0.1 to 0.59 ± 0.03 (*p* < 0.001) on the topographies (Figure 6I).

## Discussion

### Endothelial monolayer integrity is disrupted by senescent endothelial cells and loses the ability of mechanosensation and mechanotransduction

Long-term endothelialization of foreign body surfaces is a large unmet need for CVIs to improve their hemocompatibility (33). A promising approach to promoting endothelialization of artificial materials is to structure their luminal surfaces, as it enhances the biomechanical reorganization of ECs in the early stage of endothelialization (24, 34). However, while many studies have focused on the use of low-passage and proliferating HUVECs, in a clinically relevant setting of elderly patients with co-morbidities receiving CVIs, autologous endothelial progenitors and ECs required for endothelialization are most likely functionally impaired, have limited replicative capacity and enter RS (35, 36). Biochemical changes during the transition from proliferating to senescent ECs are well characterized, but associated biomechanical changes are relatively unknown. To gain further insights, we induced replicative senescence in HUVECs by extended *in vitro* culture, resulting in cells with increased SA-β-gal activity, significant formation of DNA double-strand breaks and significantly shortened relative telomere length, three typical markers of RS. These observations are consistent with previously published data (26, 27). In addition, *in vitro* cell expansion affected not only cell growth characteristics such as PDT, cell density, and replication capacity, but also cell morphology and the integrity of the EC monolayer. High-passage HUVECs lost their typical cobblestone morphology, another hallmark of senescent ECs that has also been described by others (28, 29). Next, we analyzed how a monolayer of senescent ECs behaves under physiological flow and shear stress conditions, as this is of great importance for successful endothelialization of CVIs. Physiologically in response to the laminar flow, the Golgi of ECs is located upstream of the nucleus, pointing against the flow direction (37). Furthermore, VEC protein is mainly organized along the major cell axis in the form of linear, continuous AJs between cells and is supported by cortical F-actin bundles parallel to the flow. Perpendicular to the direction of flow across the minor cell axis, VEC-AJs obtain a discontinuous, zipper-like morphology (38). In addition, the formation of F-actin stress fibers parallel to the linear AJs and localized along the edges of zipper-like AJs enhance ECs connectivity, promote EC elongation and orientation parallel to the flow, contributing to increased stress resistance (39). Both low-passage and senescent HUVECs showed counterflow polarization of the Golgi apparatus, but senescent HUVECs failed to achieve flow-dependent actin cytoskeleton organization. These findings are consistent with studies describing dramatic morphological changes due to RS or senescence induced by inflammatory stress (40, 41). There is evidence that senescent cells acquire significantly enlarged morphology due to the continued stimulation of cell growth pathways, such as mitogen-activated protein kinase and mammalian target of rapamycin pathways (42). Furthermore, RS is known to correlate with increased monolayer permeability due to impaired AJs (43). Consequently, the expansion of senescent HUVECs is associated with impaired intracellular transport of VEC protein at the site of cell-cell contacts and with reduced VEC protein expression (28) which is also shown in our study. Overall, our results support the fact that the increase in cell volume, the dysfunctional organization of AJs and the low extent of cell shape adaptation under physiological flow conditions due to RS negatively affect the ability of cells to sense mechanical cues (mechanosensation) and to transduce them into biochemical signals (mechanotransduction).

### Gratings of 1 μm height and width restore the monolayer integrity in senescent human umbilical cord vein endothelial cell monolayers

Several studies have proposed that surface modulation with defined periodic topographies can have great potential for stabilizing healthy EC monolayers under static and flow conditions (11, 24, 33). In fact, there is a positive impact of anisotropic-pattered topographies with gratings on cell-surface and cell-cell interactions as well as cell shape adaptation during endothelialization (44, 45). For example, Gorji et al. showed that ECs cultivated under static conditions on a micro-scale grating topography respond similarly to the AJs and actin cytoskeleton remodeling that occurs under laminar flow conditions. In particular, EC cultivation on gratings promote their shape elongation due to F-actin stress fibers and the formation of linear VEC AJs, as shown by the increase in EC aspect ratio and tortuosity index, respectively (46). In line with these results, Franco et al. demonstrated a 40% increase in endothelial spreading on gratings with groove width and depth of 1 μm due to focal adhesion kinase (FAK) activation, as well as enhanced FA maturation and myosin-II dependent cell contractility (47). Robotti et al. showed that HUVECs retained their monolayer connectivity and AJ integrity under flow conditions when cultured on micro-sized gratings with 1 μm height and width under both physiological WSS values (1.4 Pa) as well as supra-physiological WSS values of LVAD luminal surfaces (up to 10 Pa) (24). While our data confirm these observations in low-passage HUVECs, we also show for the first time that gratings with 1 μm height and width improve essential biomechanical properties such as maturation of FAs and AJs, as well as cell elongation and alignment in senescent HUVECs.

### Microtopographical guidance on a surface with gratings increases yes-associated protein nuclear translocation

Previous works have shown that surface modulation may impact cellular behavior beyond cell adhesion and biomechanical organization (48). In fact, topography also dictates the spatial organization and maturation of FAs, which promotes the assembly and activation of multiple proteins. For example, FAK-mediated paxillin phosphorylation induces the recruitment of vinculin at the adhesion site, which is responsible for the formation of actin stress fibers and enhanced cell elongation (49). These observations are consistent with our data showing significant FA maturation and reorganization of actin stress fibers after cultivation on gratings. Recent studies have also highlighted the involvement of YAP, a key mechanosensor and mechanotransducer of the Hippo signaling pathway that plays a critical role in correlating external mechanical stimuli with changes in gene expression. More specifically when the Hippo pathway is active, YAP becomes phosphorylated, resulting in cytoplasmic retention. However, when the Hippo pathway is inactive, YAP translocates into the nucleus and promotes cell proliferation (50, 51). It was previously reported that in confluent EC monolayers the VEC protein retains YAP in the cytoplasm by interacting with its cytoplasmic tail (52, 53). However, AJs do not simply provide sites for YAP inactivation, but they can further modulate Hippo pathway activity in response to mechanical stress signals (54). In the present study, when grown on a flat surface, ECs with cobblestone shape formed mature monolayers characterized by discontinuous, zipper-like AJs and junctional actin (55). In contrast, ECs cultivated on gratings exhibited an elongated shape, continuous, linear AJs and a strong YAP nuclear translocation.

This finding can be explained by the fact that the engagement of integrin on the grating surface has been proved to relocalize YAP in the nucleus, supporting the hypothesis that after cultivation on gratings, FA maturation is responsible for enhanced YAP shuttling in the nucleus (25). Parallel, the spatial reorganization of F-actin filaments in form of elongated stress fibers as well as the VEC protein in form of linear VEC AJs may also be in part responsible for the YAP nuclear translocation (56). In conclusion, EC shape elongation due to FA maturation and the formation of actin stress fibers and linear AJs are mainly responsible for the enhanced YAP shuttling in the nucleus.

### The gratings improve the proliferative capacity of endothelial cells without inducing an early onset of senescence

Based on the aforementioned observations, we hypothesized that modulating the surface topography could aid senescent cells not only improve their cytoskeletal organization, but also increase their proliferation rate and even enhance their replicative capacity. In a study that compared the impact of patterned anisotropic topographies with different groove sizes decreasing in width from 50 to 0.5 μm revealed a significant increase in cell proliferation after cultivation on gratings over a total period of 5 days relative to the flat surface (57). In our study, we observed an improved proliferation rate of ECs on anisotropic topographies, as evidenced by EdU staining after 16 h. In addition, we found that long-term cultivation of ECs on a surface with gratings has a positive impact on the overall replicative capacity and the proliferation rate, while it did not lead to an increase in DNA damage, SA-β-gal accumulation or telomere shortening.

## Conclusion and translational implications

In summary, we confirmed the negative effects of RS on the formation of a stable and mature EC monolayer. In addition, we found that extended *in vitro* cell expansion on micro-structured surfaces with gratings improves the impaired integrity of senescent endothelial monolayers and increases their proliferative capacity without inducing an early onset of senescence. This is a new and important insight into the field of endothelial senescence and could represent a potential strategy to improve the endothelialization of foreign surfaces and biointegration. Indeed, the clinical translation of such topographies to luminal surfaces is feasible from both a manufacturing and regulatory perspective, thus the implementation of specific textures on cardiovascular devices could be beneficial.

## Limitations

Senescence protects against malignant transformation because it coordinates tissue morphogenesis and prevents further proliferation of dysfunctional cells. Consequently, reversal of senescence can increase the risk of tumor growth. In addition, given the heterogeneity of senescent EC monolayers, the morphological alterations that occur due to RS affect a number, but not all, of the cells of a monolayer. It is therefore possible that the increase of VEC connectivity or the decrease of tortuosity index due to AJs remodeling after cultivation on the micro-structure surface with gratings mainly occur in non-senescent ECs. Moreover, due to cytoskeletal tension after cultivation on gratings, YAP nuclear translocation may only enhance proliferation of cells that do not have the typical flattened and enlarged phenotype of senescent cells. We have shown that senescent cells exhibit a significantly increased YAP nuclear signaling when cultured on a flat surface, but this did not affect their proliferation capacity. Future studies are needed to further investigate the rejuvenating impact of anisotropic topographic structures. It is well known that proteins in the culture medium play a key role in mediating cell-surface interactions. This may be important in the case of ECs interacting with flat vs. textured surfaces. This suggests that the positive impact of the gratings on EC proliferation may only result from increased protein adsorption and enhanced protein-substrate interaction, leading to further stabilization of the monolayer. Further studies to quantify protein adsorption are needed.

## Data availability statement

The raw data supporting the conclusions of this article will be made available by the authors, without undue reservation.

## Ethics statement

The studies involving human participants were reviewed and approved by the Institutional Ethics Board of Charité—Universitätsmedizin Berlin (EA2/178/13). The patients/participants provided their written informed consent to participate in this study.

## Author contributions

TN-S, CG, AF, NCh, and ME: scientific oversight. TN-S, VE, CG, and AF: experimental design. VE, NCe, TN-S, CG, HM, KK, HR, SM, FP, and FK: experimentation. VE, TN-S, and SN: manuscript writing. All authors: interpretation of data and critical revision.

## Abbreviations

AJ: adherens junction
AT: anisotropic topography
BSA: bovine serum albumin
CI: connectivity index
COC: cyclic olefin copolymer
CV: cardiovascular
CVI: cardiovascular implant
DAPI: 4’,6-diamidino-2-phenylindole
EC: endothelial cell
EdU: ethynyl-2’-deoxyuridine
FA: focal adhesion
HUMSC: human umbilical cord mesenchymal stems cell
HUVEC: human umbilical cord vein endothelial cell
LVAD: left ventricular assist device
RS: replicative senescence
PBS: phosphate-buffered saline
PCR: polymerase chain reaction
PD: population doubling
PDT: population doubling time
PDMS: polydimethylsiloxane
PFA: paraformaldehyde
SA-β-gal: senescence-associated β-galactosidase
VEC: vascular endothelial cadherin
WSS: wall shear stress
YAP: yes-associated protein.
