# Supplementary material for: Anisotropic topographies restore endothelial monolayer integrity and promote the proliferation of senescent endothelial cells
Source: Front Cardiovasc Med. 2022 Oct 5;9:953582. doi: 10.3389/fcvm.2022.953582 (PMC9579341; doi:10.3389/fcvm.2022.953582)
Supplement: Supplementary file 1 [file Data_Sheet_1.docx]

**Supporting Information**

**Anisotropic topographies restore endothelial monolayer integrity and promote the proliferation of senescent endothelial cells**

Vasileios Exarchos^a,b^, Sebastian Neuber^a,b^, Heike Meyborg^a,b^, Costanza Giampietro^c,d^, Nafsika Chala^e^, Silvia Moimas^e^, Hristian Hinkov^a,b^, Friedrich Kaufmann^a^, Francesca M. Pramotton^c,d^, Katrin Krüger ^a,b,f^, Hector Rodriguez Cetina Biefer ^a,b,g^, Nikola Cesarovic^a,h^, Dimos Poulikakos^e^, Volkmar Falk^,f,h,i^, Maximilian Y. Emmert^a,b,f,h,j^, Aldo Ferrari^c,d,e^, Timo Z. Nazari-Shafti^a,b,h^ *****

1. Cardiosurgical Research Group, Department of Cardiothoracic and Vascular Surgery, German Heart Center Berlin, 13353 Berlin, Germany
2. Translational Cardiovascular Regenerative Technologies Group, Berlin Institute of Health at Charité – Universitätsmedizin Berlin, BIH Center for Regenerative Therapies, 13353 Berlin, Germany
3. Experimental Continuum Mechanics, Empa Swiss Federal Laboratories for Materials Science and Technology, 8600 Dübendorf, Switzerland
4. Institute for Mechanical Systems, Department of Mechanical and Process Engineering, ETH Zurich, 8092 Zurich, Switzerland
5. Laboratory of Thermodynamics in Emerging Technologies, Department of Mechanical and Process Engineering, ETH Zurich, 8092 Zürich, Switzerland
6. Clinic for Cardiovascular Surgery, Charité – Universitätsmedizin Berlin, 13353 Berlin, Germany
7. Department of Cardiac Surgery, City Hospital of Zürich, Site Triemli
8. Department for Cardiovascular and Thoracic Surgery, German Heart Center Berlin, Berlin, Germany.
9. Department of Health Sciences and Technology, ETH Zurich, 8093 Zurich, Switzerland
10. Institute for Regenerative Medicine, University of Zurich, 8044 Zurich, Switzerland,
11. Berlin Institute of Health at Charité – Universitätsmedizin Berlin, BIH Biomedical Innovation Academy, BIH Charité (Junior) (Digital) Clinician Scientist Program, Charitéplatz 1, 10117 Berlin, Germany

* Corresponding author


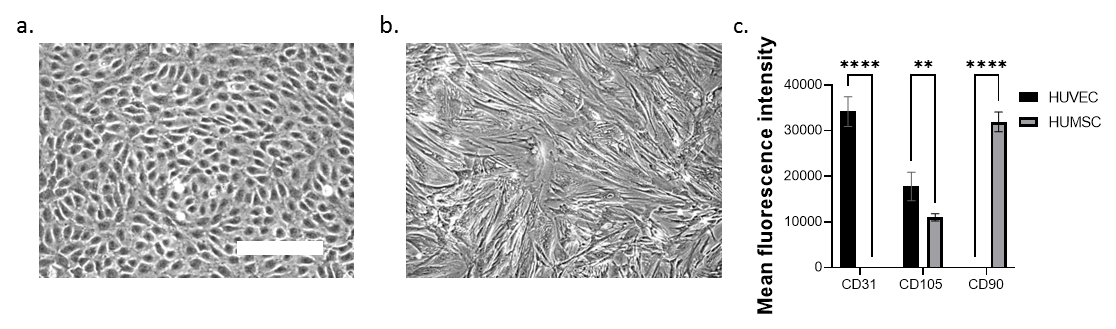
**Figure S1:** **Morphology and surface marker expression of isolated HUVECs and HUMSCs under *in vitro* conditions.**

*HUVECs isolated from fresh umbilical cords formed a confluent monolayer with a typical cobblestone morphology after 3 population doublings (PDs) under in vitro conditions (Figure S2a). They were positive for typical endothelial surface markers such as CD31 and CD105; contamination of HUVECs with spindle-shaped HUMSCs was ruled out by screening for CD90 (Figure S2b-c). (a) Phase-contrast image of a confluent HUVEC monolayer at low passage level (3 PDs) with cobblestone morphology, b. Phase-contrast image of a confluent HUMSC monolayer at low passage level (3 PDs) with spindle shape morphology, c. Mean fluorescence intensity of CD31, CD105 and CD90 for HUVECs and HUMSCs at low passage level (3 PDs) after flow cytometry analysis. Data represent the mean and standard error of the mean of 3 donors with more than 1,000 cells per condition. ****, p≤0.0001; ns, not significant. In a and b, images were taken with the same magnification; scale bar, 200 µm.*


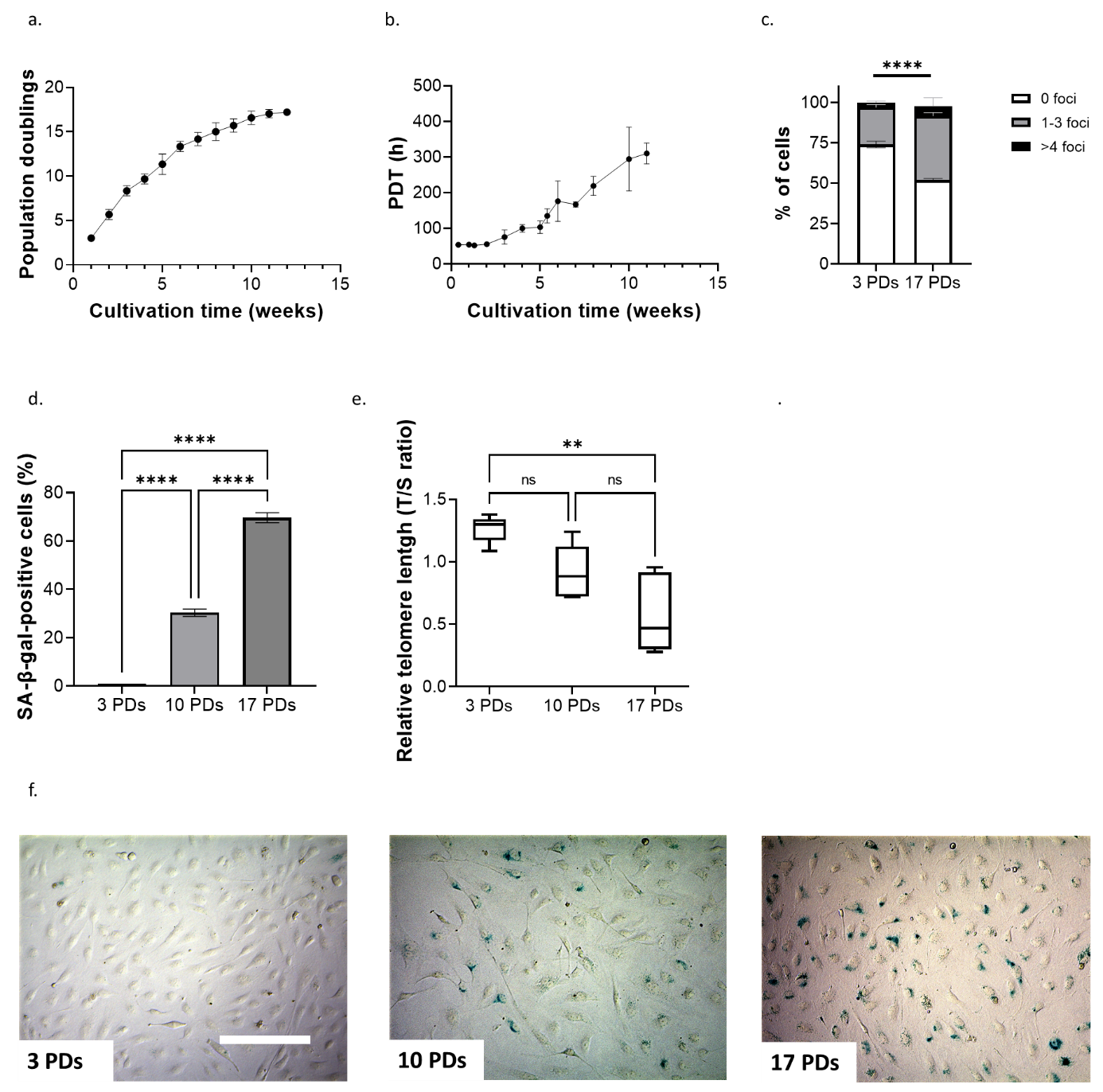


***Figure S2: In vitro expansion induce replicative senescence.***

*To induce replicative senescence (RS), HUVECs were seeded at a density of 20,000 cells/cm² on plates coated with 1.5% gelatin and passaged at 80-90% confluence until they reached RS. Cell cycle arrest was observed after a total of 14±1.4 weeks and 17±0.2 PDs (Figure S3a). Conversely, the mean PDT generally increased with an increase in the number of passages during cell expansion (Figure S3b). Senescent HUVECS expressed higher levels of phosphorylated histone H2AX (γ-H2AX), which is indicative of an increase in DNA damage: in 74±2% of HUVECS after 3 PDs and 52±1% after 17 PDs γ-H2AX foci were not detected (Figure S3c). The percentage of cells showing 1-3 γ-H2AX foci was 23±1% after 3 PDs and 40±2% after 17 PDs, and 3±1% and 6±4% of the cells had more than 4* *γ-H2AX foci after 3 and 17 PDs, respectively (Figure S3c). As expected, intracellular SA-β-gal accumulation increased over time and was 70±2% when the growth of the HUVECs reached the plateau (Figure S3d, Figure S5). In addition, relative telomere lengths of HUVECs decreased up to 50% of their initial length during in vitro expansion (Figure S3e).* *Furthermore, a progressive change in the whole monolayer architecture was observed during in vitro expansion: after 3 PDs, HUVECs showed single ovoid nuclei and a cobblestone-like morphology but were significantly enlarged and lost their ability to form high confluent monolayers after 17 PDs (Figure S3f and Figures S5a and S5b). The total cell surface of HUVECs increased from 1,180±51 µm² after 3 PDs to 4,181±138 µm² after 17 PDs. In conclusion, extensive in vitro expansion of HUVECs progressively decreased their growth characteristics, significantly increased their size and induced RS, as evidenced by increased SA-β-gal activity, significant formation of DNA double-strand breaks and significant telomere attrition. (a) Cumulative growth curve during expansion of HUVECs isolated from three different umbilical cords, (b) PDT of HUVECs during in vitro expansion, (c) Percentage of cells presenting multiple γ-H2AX foci after 3 and 17 PDs, (d) Percentage of SA-β-gal-positive cells after 3 and 17 PDs, (e) Relative telomere length in relation to the number of PDs of HUVECs during in vitro expansion, (f) Representative light microscopy images of HUVECs during expansion stained for SA-β-gal (green-blue) after 3 (left), 10 (middle) and 17 (right) PDs. Cell density measured at the end of each passage when the monolayer was 80-90% confluent. Scale bar, 200 µm. In (a–e)* *data represent the mean and standard error of the mean off 3 donors with more than 275 cells per condition for the analysis of DNA damage, more than 2,800 cells per condition for the analysis of SA-β-gal activity, more than 100,000 cells per condition for the relative telomere length measurement and more than 2,800 cells per condition for the cell area measurement. **, p≤0.01; ***, p≤0.001; ****,* *p≤0.0001; ns, not significant.*


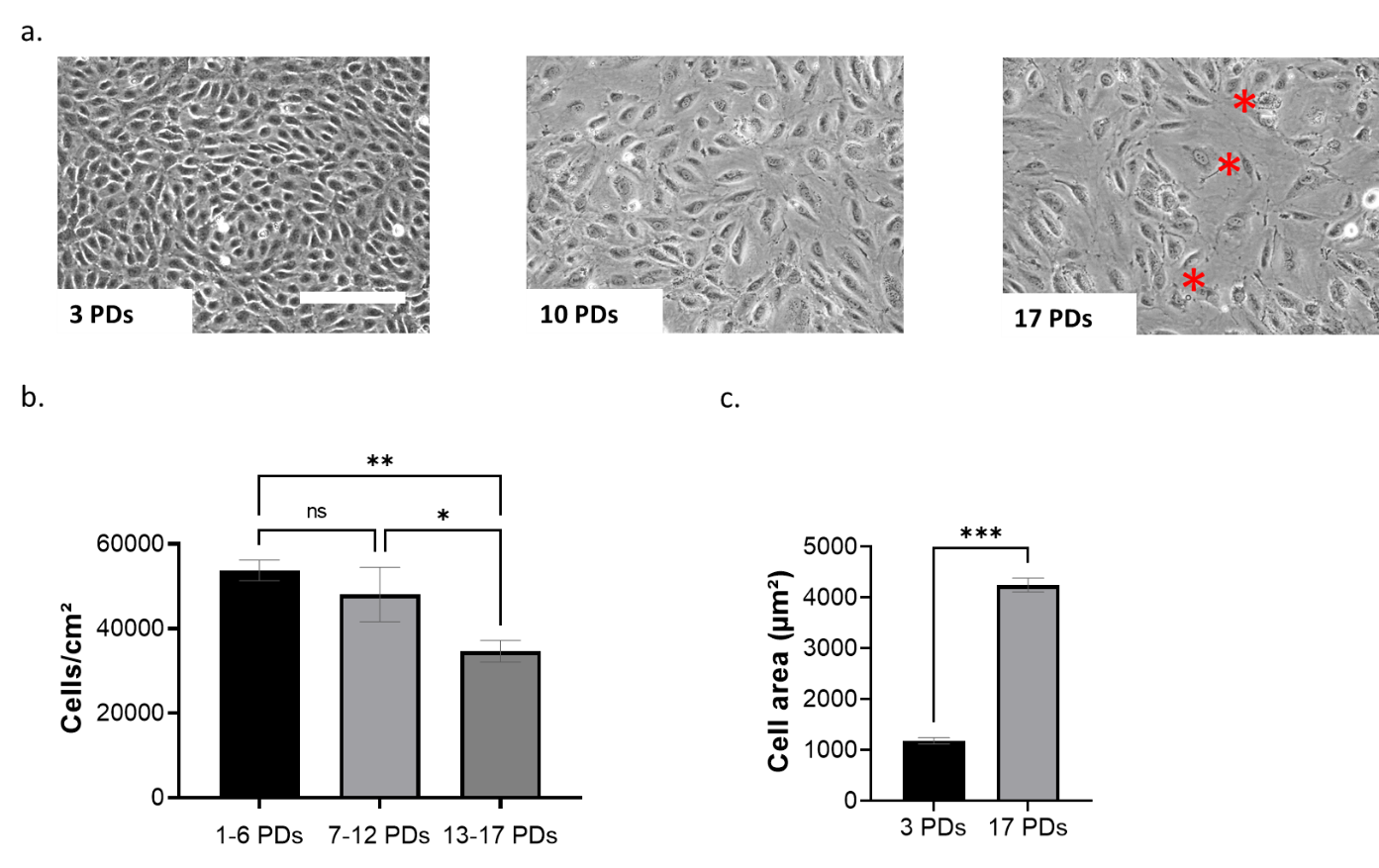


**Figure S3: Morphological alterations of HUVECs during in vitro expansion.**

*(a) Representative light microscopy images of HUVECs after 3, 10 and 17 PDs show progressive increase of the cell size during in vitro expansion. Red asterisks show sites of monolayers disruption. Scale bar, 200 µm. (b) Cell density measured at the end of each passage when the monolayer was 80-90% confluent. (c) Progressive increase of the cell size during in vitro expansion. Data represent the mean and error bars of 3 donors. *, p<0.05; **, p≤0.01; ***, p≤0.001; ns, not significant.*

**
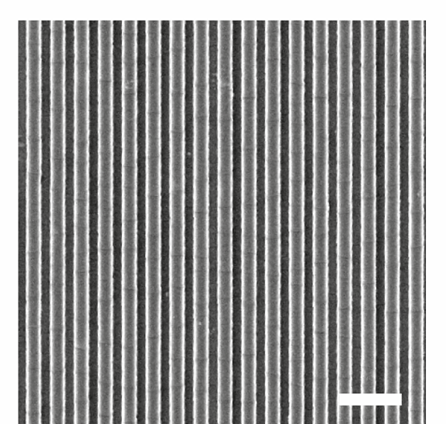
**

**Figure S4: Surface with gratings of 1 µm depth and height.**

*For the scanning electron microscope images, the specimens were sputter-coated with gold/palladium using a BAL-TEC SCD-050 sputter coater and imaged with a Zeiss ULTRA 55 SE. Scale bar, 5 µm. Measurements of gratings showed mean grating size of 0.96 ±0.6 µm*
